# Analysis of an obstetrics point-of-care ultrasound training program for healthcare practitioners in Zanzibar, Tanzania

**DOI:** 10.1186/s13089-021-00220-y

**Published:** 2021-04-08

**Authors:** Elizabeth A. Hall, Danielle Matilsky, Rachel Zang, Naomasa Hase, Ali Habibu Ali, Patricia C. Henwood, Anthony J. Dean

**Affiliations:** 1 Department of Emergency Medicine, Providence St. John’s Health Center, 2121 Santa Monica Blvd, Santa Monica, CA 90404 USA; 2 Point-of-Care Ultrasound in Resource-Limited Environments (PURE), Malvern, PA USA; 3 Department of Emergency Medicine, Howard County General Hospital, John Hopkins University, Baltimore, MD USA; 4 Department of Emergency Medicine, University of Pennsylvania, Philadelphia, PA USA; 5 Obstetrics & Gynecology, Wayne State University, Detroit, MI USA; 6 Chake Chake Hospital, Pemba, Zanzibar, Tanzania; 7 Department of Emergency Medicine, Thomas Jefferson University Hospital, Philadelphia, PA USA; 8 Emergency Medicine and Emergency Medicine in Radiology, University of Pennsylvania, Philadelphia, PA USA; 9 World Interactive Network for Focused Ultrasound (WINFOCUS), Philadelphia, PA USA

**Keywords:** Obstetrics, Ultrasonography, Global health, Medical education, Zanzibar, Antenatal health providers, Clinician-performed ultrasonography

## Abstract

**Background:**

A point-of-care ultrasound education program in obstetrics was developed to train antenatal healthcare practitioners in rural Zanzibar. The study group consisted of 13 practitioners with different training backgrounds: physicians, clinical officers, and nurse/midwives. Trainees received an intensive 2-week antenatal ultrasound course consisting of lectures and hands-on practice followed by 6 months of direct supervision of hands-on scanning and bedside education in their clinical practice environments. Trainees were given a pre-course written exam, a final exam at course completion, and practical exams at 19 and 27 weeks. Trainees were expected to complete written documentation and record ultrasound images of at least 75 proctored ultrasounds. The objective of this study was prospectively to analyze the success of a longitudinal point-of-care ultrasound training program for antepartum obstetrical care providers in Zanzibar.

**Results:**

During the 6-month course, trainees completed 1338 ultrasound exams (average 99 exams per trainee with a range of 42–128 and median of 109). Written exam scores improved from a mean of 33.7% (95% CI 28.6–38.8%) at pre-course assessment to 77.5% (95% CI 71–84%) at course completion (*P* < 0.0001). Practical exam mean scores improved from 71.2% at course midpoint (95% CI 62.3–80.1%) to 84.7% at course completion (95% Cl 78.5–90.8%) (*P* < 0.0005). Eight of the 13 trainees completed all training requirements including 75 proctored ultrasound exams.

**Conclusion:**

Trainees improved significantly on all measures after the training program. 62% of the participants completed all requirements. This relatively low completion rate reflects the challenges of establishing ultrasound capacity in this type of setting. Further study is needed to determine trainees’ long-term retention of ultrasound skills and the impact of the program on clinical practice and health outcomes.

## Background

Maternal and fetal health are of global concern due to the high burden of morbidity and mortality borne by these groups. In Zanzibar, which is a semi-autonomous part of the United Republic of Tanzania, a pregnant woman has an approximately 1/200 chance of dying from her condition (maternal mortality rate of 545 per 100,000 live births) and there is a > 1/20 chance of her child dying in infancy (infant mortality rate of 61 per 1000 live births) [1, 2]. In comparison, in industrialized nations, maternal mortality rates are typically less than 10 per 100,000 live births and infant mortality rates are around 5 per 1000 live births [3]. Zanzibar has its own Ministry of Health and Social Welfare which is working on strategies to decrease infant and maternal mortality in the country [4]. Recent initiatives have included improving access to health facilities with skilled birth attendants and prenatal ultrasound imaging.

Ultrasound is a safe and non-invasive tool that has wide-ranging uses in the evaluation and management of pregnancy. Through sonography, it is possible to obtain an estimation of gestational age (and thereby estimated date of parturition and intrauterine growth restriction), to detect fetal anomalies, identify multiple gestations, abnormal fetal lie, and risk factors for complicated delivery or need for specialized perinatal care of the infant. In an environment in which home deliveries (with or without a midwife) are the norm and resources are inadequate for widespread hospitalization for childbirth, this information may allow for improved planning of deliveries both at home and those needing referral to locations with more advanced obstetrical services. Since roads and transportation infrastructure are limited, travel to a referral hospital requires planning and allocation of resources. In addition to those already mentioned, the goals of the scan include reduction of induction of labor for post-term pregnancy and improvement in a woman’s pregnancy experience [5].

Despite its demonstrated benefits, many pregnant women in rural communities do not have access to antenatal ultrasound [6–8]. One of the major impediments is that it is often only available at ultrasound imaging suites located in hospital-based radiology departments and whose immobile equipment require a constant electrical supply. Furthermore, it is a timely two-step process with images obtained by technologists with subsequent review by imaging specialists who were not primarily caring for the patient, and whose consultant reports often take several days to reach the patient’s primary providers, leading to delays in diagnosis and decision-making [9]. Travelling to such sites often entails arduous time-consuming journeys as well as other personal and financial burdens for pregnant women. Clinician-performed ultrasonography (CPUS, also referred to as “point-of-care ultrasound”) puts modern ultrasound equipment, which is robust, portable, inexpensive, and increasingly user-friendly, directly into the hands of practicing providers. Patients can receive an ultrasound in their local primary care clinic with immediate results, decision-making, and arrangement for next steps. Several studies have demonstrated the utility of a variety of applications of CPUS in limited-resource settings [10–15]. In addition to the WHO recommendations, several studies have demonstrated the value of CPUS in antenatal care [16–18].

A significant challenge to the implementation of CPUS is in the development of ultrasound skills on the part of the providers so they can make use of this technology. These skills include psychomotor components, cognitive training, and new clinical skills that integrate the ultrasound into new patterns of decision-making. No scientific study has definitively identified the minimum time required for trainees to assimilate the complex cognitive, clinical, psychomotor skills required for ultrasound; however, expert consensus opinion and reports of training programs in developing world settings suggest that learning antenatal ultrasound requires more than a few days of classroom teaching and practice on healthy models [12, 14, 17, 19]. There is an additional challenge for providers in independent professional practices: new skill sets must be mastered and new practice patterns assimilated in the midst of busy clinical schedules. To address these challenges, CPUS training programs for adult learners ideally utilize a longitudinal approach with direct feed-back and proctoring within the trainees’ practice setting [20]. Several models of longitudinal training have been described for trainees with a variety of practice settings and educational backgrounds [12–14, 17, 18, 20–23]. The current paper describes a training program for antenatal health providers with three different levels of professional training in the rural resource-limited island of Pemba in Zanzibar, Tanzania.

## Methods

In 2015 and 2016 at the invitation of Zanzibar’s Ministry of Health (MOH), a needs assessment was completed by the PURE organization (Point-of-care Ultrasound in Resource-limited Environments), an American-based non-governmental organization, to evaluate the ultrasound capacity at eight rural and district hospitals in Zanzibar. Based on this needs assessment reviewed by the MOH, it was collectively determined that the most useful modality for focused ultrasound training would be in antenatal and obstetrical evaluations. PURE designed a CPUS training program for antenatal health providers in Pemba Island of Zanzibar per request of the MOH. This paper reports on the prospective observational findings regarding the training program. Trainees and training sites were selected by the MOH based on hospital and community needs, trainees’ interest, and proficiency in English.

### Setting and hospital resources

The health system in Zanzibar uses a “hub and spokes” model with four levels of care. The most basic level is the Primary Health Care Unit (PHCU) which is staffed by nurse-midwives and provides basic care, health monitoring, antenatal, home delivery, and health education services. Some PHCUs have on-site laboratory and delivery services and are specified as “PHCU+”. Both types of PHCU refer more complex medical problems to either Public Health Care Centers (PHCC) or District Hospitals. There is a single tertiary care referral hospital located on the southern island of Unguja (Mnazi Mmoja Hospital). On the northern island of Pemba which has about 30% of the Zanzibar landmass (950 square miles) and population (1.3 million), there are 58 PHCU, two PHCC, and three district hospitals each with about 100 beds [4, 8, 23]. The three facilities involved in the training program included Chake and Wete District Hospitals, and the PHCC at Micheweni. The two district hospitals provide delivery, laboratory, and pharmacy services and have the capacity to perform basic surgery including cesarean sections. The hospitals each have one ultrasound machine and an ultrasound technician that is shared among all departments available during working hours. Micheweni was chosen because it serves a large catchment area in Pemba and is designated to become a district hospital and at the time of the study had no radiology services.

### Training design and protocol

The initial classroom-based ultrasound training course was provided by four residency-trained emergency physicians (EPs) and two specialists in obstetrics and gynecology (Ob/Gyn). Two of the EPs had completed ultrasound fellowships and one was in the process of fellowship. One of the EPs was a senior resident with focused training in ultrasonography [24–26].

The trainee group included midwives, clinical officers, and physicians (see “Results” for details). All trainees agreed to participate in the study and provided written consent. The initial 2-week (5 days per week) training course included ultrasound basics, physics, and core obstetric applications based on the *WHO Recommendations on Antenatal Care for a Positive Pregnancy Experience: Ultrasound Examination* and *Ultrasound in Obstetrics and Gynecology: A Practical Approach* [5, 27]. More than 50% of the time each day was dedicated to scanning practice on volunteer models. Prior to training, all participants completed a written pre-test to determine their level of knowledge and use of obstetrical ultrasound. The pre-test was composed of 8 multiple choice questions based on material taught during the course. In order to ensure the participants could focus on the training without distractions, the MOH excused all clinical duties during the initial training period and provided them with salary support. The study protocol was approved by the local Institutional Review Board, the Zanzibar Medical Research and Ethics Committee (ZAMREC) and the University of Pennsylvania.

The initial course was followed by 6 months of longitudinal supervision and training during which trainees obtained obstetric ultrasounds in their clinical practice environments. An ultrasound trainer was continually present in Zanzibar to provide direct proctoring and/or review of recorded exams from the previous few days as s/he traveled to the training sites on a rotating basis, so that each week two sites received two visits and one of the sites received a single visit. Each participant had access to between 6 and 7 h of in-person training per week, but individual training time varied based on a variety of uncontrolled factors. These included the number of patients needing ultrasound, the number of patients waiting to be seen in the clinic, the number of trainers at the site during training sessions, personal motivation, and the number of scans being done by other trainees (machine access). Trainees recorded de-identified images and video clips of their exams. Information obtained from the ultrasound exam was given to the patient and (if different from the sonologist) the medical team involved in the patient’s care. Inconclusive exams or those about which the trainee was uncertain were documented. All ultrasound exams were reviewed for adequacy of interpretation and technical quality by the local trainer during the rotating site visits. The imaging goals of the ultrasound exams (see Table 1 for details) were based on the recommendations of the *AIUM-ACR-ACOG-SMFM-SRU Practice Parameter for the Performance of Standard Diagnostic Obstetric Ultrasound Exams* [28]. During their site visits, trainers provided lectures and didactic sessions as well as ultrasound case reviews. Trainees had the option of contacting a trainer by telephone for further assistance but local internet services and computer resources did not support transmission of images in real-time.

**Table 1:** Training requirements for PURE pregnancy screening and obstetrics ultrasound course completion

● Received 30 h of theoretical teaching covering the following topics
○ Ultrasound physics, technology
○ Estimation of gestation age: crown–rump length, femur length, biparietal diameter, abdominal circumference, head circumference
○ Multiple gestation
○ Fetal presentation and lie
○ Normal fetal anatomy
○ Placenta location, identification of placenta previa
○ Amniotic fluid index
○ Focused assessment for free fluid (FAFF) in the peritoneum
○ Ultrasound program administration and quality assessment and control
○ Ultrasound safety, machine maintenance, infection control
● Performed a minimum of 75 obstetric ultrasound exams under supervision
● Performed satisfactory (minimum 75% correct) on a written exam covering the theoretical knowledge listed above
● Performed satisfactory (minimum 85% correct) on a practical skills assessment of the following
○ Performs a systematic pelvic ultrasound exam
○ Optimizes and correctly orientates ultrasound images
○ Correctly identifies an intrauterine pregnancy
○ Can identify fetal number
○ Can recognize fetal presentation and lie
○ Can identify placental location
○ Can determine fetal heart rate and identifies fetal distress
○ Obtains accurate measurements of the fetal biparietal diameter, head circumference, abdominal circumference, and femur length
○ Fetal dating and/or weight estimation
○ Amniotic fluid index and recognition of polyhydramnios and oligohydramnios
○ Identifies common obstetric abnormalities such as molar pregnancy, placenta previa, and intrauterine fetal demise and has knowledge of their management
○ Performs a focused assessment for free fluid in the correct clinical context

Trainees completed a mid-course objective structured clinical examination (OSCE) at 17 weeks to monitor their psychomotor skills, image acquisition, and accuracy of interpretation. An OSCE was not provided at the beginning of the course as the majority of trainees had no prior experience with ultrasound. At the conclusion of the 6-month training course, an OSCE and written examination were completed at 27 weeks. The OSCE was scored using a standardized rating system previously developed and deployed in other settings [20]. The written exam was composed of 28 multiple choice questions including the 8 from the pre-test and required a passing score of greater than 75%.

*While the content of the course was defined *a priori*, criteria for successful completion were outlined in anticipation that they might need to be modified based on the performance of the trainees since there is no established standard as to what should be included on an antenatal PoCUS exam* [5, 20, 22, 29]. This arrangement reflects the goals of this study since there is little data about what ultrasound skills can be mastered by trainees such as ours in this healthcare setting. The initial goal for successful course completion was a passing score of 75% on the written exam, successful performance of the OSCE with a passing score of 85%, and completion of at least 75 adequate obstetric ultrasound exams. The American College of Emergency Physicians’ policy statement on ultrasound competency recommends a minimum of 25–50 high-quality exams performed per application [24]. Thus performing a minimum of 75 proctored exams was chosen to increase the odds of obtaining competency and was thought to be easily achieved during the 6-month course. Similarly, it was observed that many trainees struggled with femur identification and measurement. For this reason, this skill was excluded from the requirements for the OSCE and for course completion. The details of the curriculum and ultrasound applications taught are listed in Table 1 along with the initially proposed completion requirements for the course.

### Ultrasound equipment

Three SonoSite M-Turbo portable ultrasound machines (Sonosite Inc., Bothell, WA, USA) were used for the study, one at each hospital site. Each machine was equipped with an extra battery, curved array abdominal probe (5–2 MHz), and linear array probe (5–10 MHz). Endovaginal ultrasonography was not included in the course. Each machine had software to perform prenatal calculations, as well as for storage of ultrasound images and clips.

### Statistical analysis

The primary outcome of this study was trainees’ performance on a final OSCE and written exams compared to their initial written and practical assessment as well as successful completion of the training program. We also analyzed trainees’ educational background with their performance and number of completed exams. Results are reported as means with 95% confidence intervals, and medians, where applicable. Paired *t*-testing was used to compare means. All analyses were completed using PRISM (Graphpad Software Inc., version 7.0d).

## Results

A total of 15 participants, composed of physicians, clinical officers, and nurses/midwives, were recruited to enroll in the training program (Table 2). The trainees were all full-time MOH employees affiliated with the obstetrics and gynecology departments at the participating facilities. A majority of the trainees were nurses/midwives. Over the 6 months of the course, two trainees dropped-out, one due to a lack of attendance, and the other due to a job transfer. 85% of the trainees had no prior ultrasound education and none were routinely performing ultrasound as a part of their practice. The trainees performed most of their ultrasound exams during obstetric clinics and on obstetric ward rounds. Patients included those from nearby villages as well as those referred from PHCUs.

**Table 2:** Demographic details about the trainees who completed the ultrasound training program

Demographics	Number of trainees (%)
Male	4 (30)
Female	9 (70)
District hospital [2]	8 (62)
Public Health Care Center	5 (38)
Education	
Midwife/nurse	8 (62)
Clinical officer	3 (23)
Medical officer/physician	2 (15)
Prior ultrasound experience	
None	11 (85)
Informal (experience)	1 (7.5)
Formal (education)	1 (7.5)

Thirteen trainees completed the 6-month training program. A total of 1338 proctored ultrasound exams were performed with a trainee average of 99 exams (range 42–128) and a median of 109. Most of the exams were performed during the second or third trimesters, although the clinics are open to any pregnant patient, so there were a small number of first trimester exams. Table 3 summarizes the pre- and post-training exam scores (*P* < 0.0001). Final written exam scores by professional role were nurses/midwives 73.7 ± 13.5%, clinical officers 80.9 ± 5.4%, and medical officers/physicians 87.5 ± 2.5%.

**Table 3:** Summary of written exam scores for 13 trainees pre- and post-training

	Pre-training	Post-training
Num. exam questions	8	28
Median correct answers	37.5%	82.1%
Mean correct answers	33.7%	77.5%
SD	9.4%	11.9%
95% CI of mean	28.6–38.8%	71–84%
Passed (*n*)	0	77% (10)
Failed (*n*)	100% (13)	33% (3)

A passing score was ≥ 75% correct answers

**Fig. 1:**
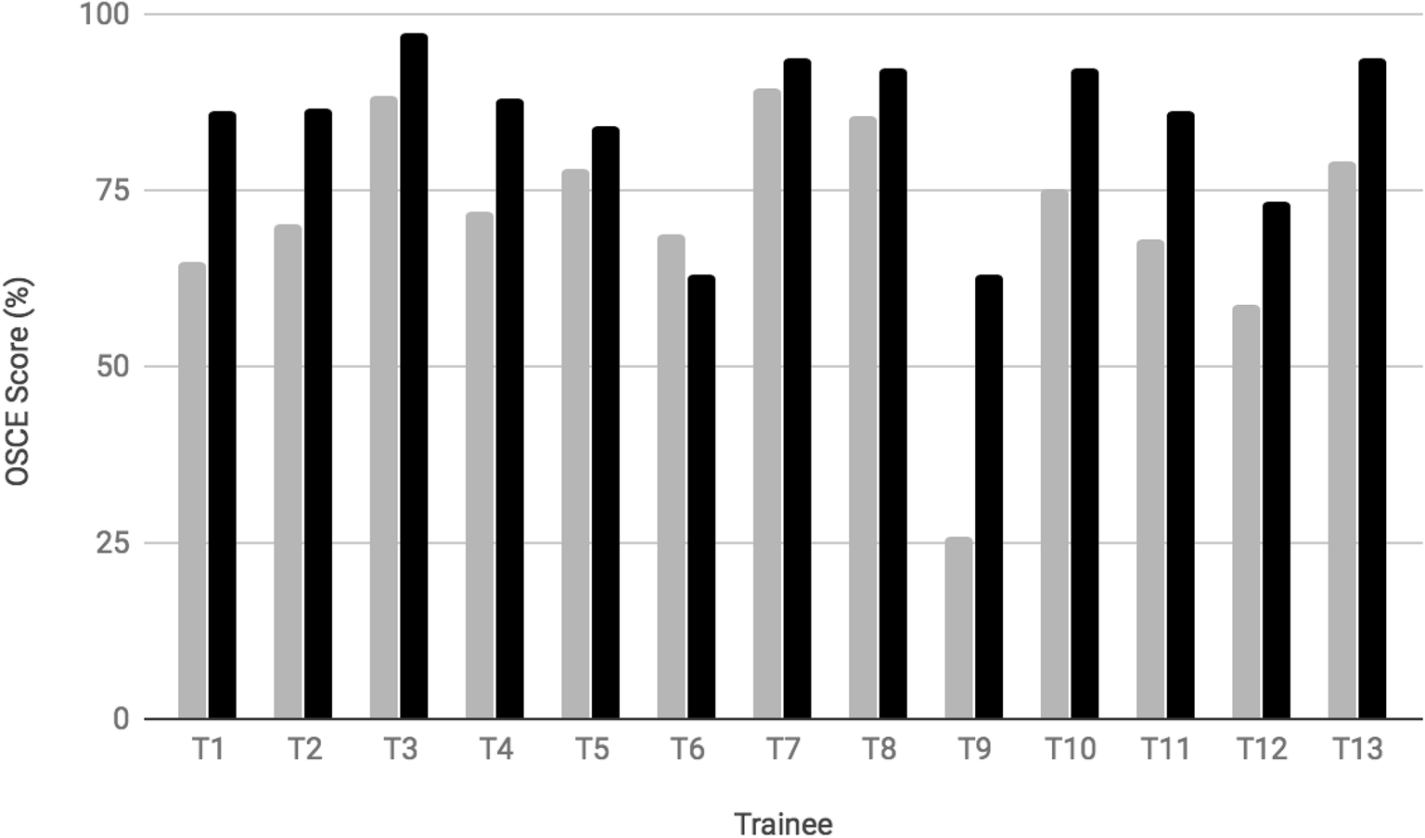
Individual OSCE scores at the mid-course evaluation (gray) and final course evaluation (black)

Each of the 13 trainees completed an OSCE at 19 weeks and at the course conclusion at 27 weeks (Table 4). Overall the mean scores increased from 71.2% (95% CI 62.3–80.1%) to 84.7% at course completion (95% Cl 78.5–90.8%) between 19 and 27 weeks (*P* < 0.0005). Figure 1 demonstrates individual trainees’ performances on the OSCE at mid-course compared to a final exam. Final OSCE exam scores by professional training were nurses/midwives 79.7 ± 11.2%, clinical officers 92.7 ± 5.6%, and medical officers/physicians 93 ± 1%.

**Table 4:** Summary of OSCE exam scores for trainees at mid-course (19 weeks) and final exam (27 weeks)

OSCE exam	Mid-course	Final exam
Median	72.1%	84.7%
Mean	71.1%	86.7%
SD	16.5%	11.1%
95% CI of mean	62.8–80%	78.5–90.8%
Passed (*n*)	23.1% (3)	46.2% (6)
Failed (*n*)	76.9% (10)	53.8% (7)

A passing score was ≥ 85% correct

**Fig. 2:**
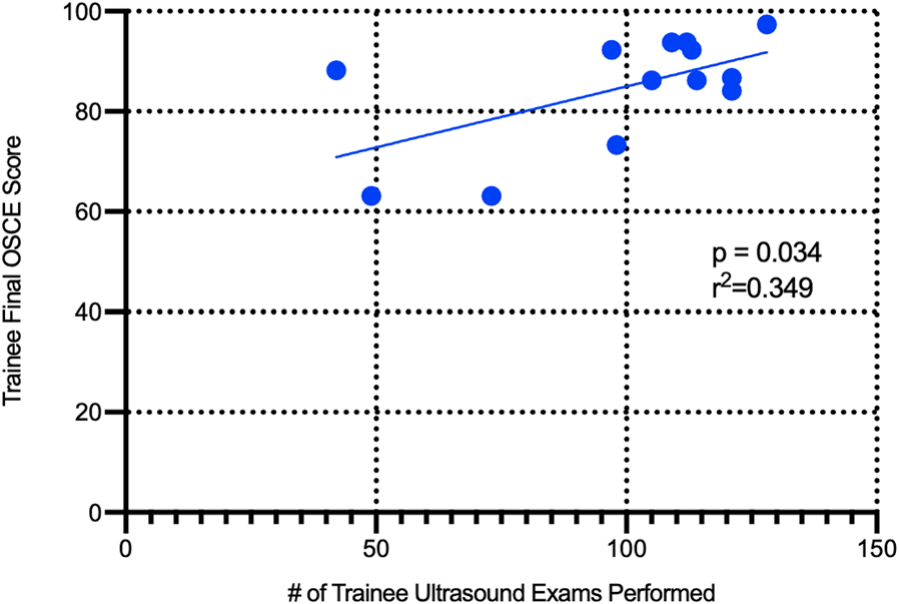
Total number of ultrasound exams performed compared to individual OSCE scores

A total of 8 (62%) of the 13 trainees successfully fulfilled the requirements for course completion: performing greater than 75 proctored ultrasound exams and passing the written exam and OSCE. This group included 2 physicians (100% success rate), 3 clinical officers (100% success rate), and 3 nurse/midwife (37.5% success rate) (Table 5). Successful trainees had a higher ultrasound exam total compared to their peers 112 ± 9.4 compared to those who did not pass 76.6 ± 33.2. Figure 2 is a scatter plot comparing final OSCE scores to the number of ultrasound exams performed and indicates a weak correlation between successful completion of OSCEs and number of scans performed (*p* = 0.034, *r*^2^ = 0.349).

**Table 5:** Summary of passing scores and completion of program requirements by trainee education

Trainee education	Final written exam	Final OSCE	Completion of all program requirements
Midwife/nurse (*n* = 8)	5 (62.5%)	4 (50%)	3 (37.5%)
Clinical officer (*n* = 3)	3 (100%)	3 (100%)	3 (100%)
Medical officer/physician (*n* = 2)	2 (100%)	2 (100%)	2 (100%)

## Discussion

By means of a program in a rural health system in Zanzibar, 62% of antenatal providers of varied medical backgrounds were trained to provide screening ultrasound exams on pregnant patients. Trainees were able to perform all tasks recommended by the WHO in limited-resource developing world settings [30]. To reflect regional practice realities, the majority of clinician-trainees were neither medical officers (general practice physicians) nor consultants (obstetrics/gynecology specialists). These findings extend the utility of this type of training which has been successfully applied in previous studies of physician providers to environments where non-physician providers are used for primary care and basic evaluations [12, 14, 22]. Six of the eight trainees who successfully completed the course were non-physicians. Thus by including non-physician providers, we increased the number of ultrasound-trained practitioners and provided one regional hospital with professionals with ultrasound skills on site. All 13 trainees significantly improved their ultrasound skills from their baseline pre-training test.

Recommendations for universal performance of an antenatal ultrasound evaluation are based on the clinically important information that it provides [5, 29]. In addition to facilitating recognition of fetal growth restriction, a major cause of perinatal morbidity and mortality, ultrasound allows improved decision-making about the level of obstetrical care that will be needed at delivery. In rural and low-resource settings, this determines the need for and timing of transfer of patients to facilities with more advanced resources. This decision is consequential first because failure to recognize a serious condition requiring transfer (e.g., placenta previa) places both mother and child at risk of death or life-long morbidity, and second because unnecessary or untimely transfers burden an already-overloaded referral system and may lead to financial and logistical burdens in an environment where many patients live far from roads and vehicular transport is limited.

While defined standards are needed for any diagnostic test one of the essential characteristics of PoCUS and CPUS is that it is a *focused* and *limited* exam. Because PoCUS is performed by providers with many other clinical duties and responsibilities, a common determinant of the *focus and limitations* of the exam is its complexity. In general, PoCUS applications tend to be more easily incorporated into practice if they are simple to perform and interpret. With respect to image acquisition, this involves sonographically accessible anatomy, easily identifiable structures, readily obtainable imaging planes, and key findings that are subject to binary (“yes/no”) interpretation (with “I don’t know” always being a third option). In our study, we found that measurement of femur length was challenging to many learners. This is most likely because accurate femur acquisition relies on an accessible fetal position, can require more difficult imaging planes, and can be confused as the humerus bone [31]. Accordingly, we did not include it as a requirement for successful completion of the ultrasound program. This will result in a minor limitation in the estimation of fetal age for trainees of this course since in the third trimester methods that combine several measurements are slightly more accurate than those that use one only [28, 29, 32]. However, all methods of estimating due date are most accurate in the first trimester with declining accuracy thereafter and a margin of error of 3 weeks in the third trimester [32–35]. This limitation would not affect the utility of the exam to determine multiple gestations, fetal lie, placenta previa, and oligo- or polyhydramnios, typically in the third trimester.

**Fig. 3:**
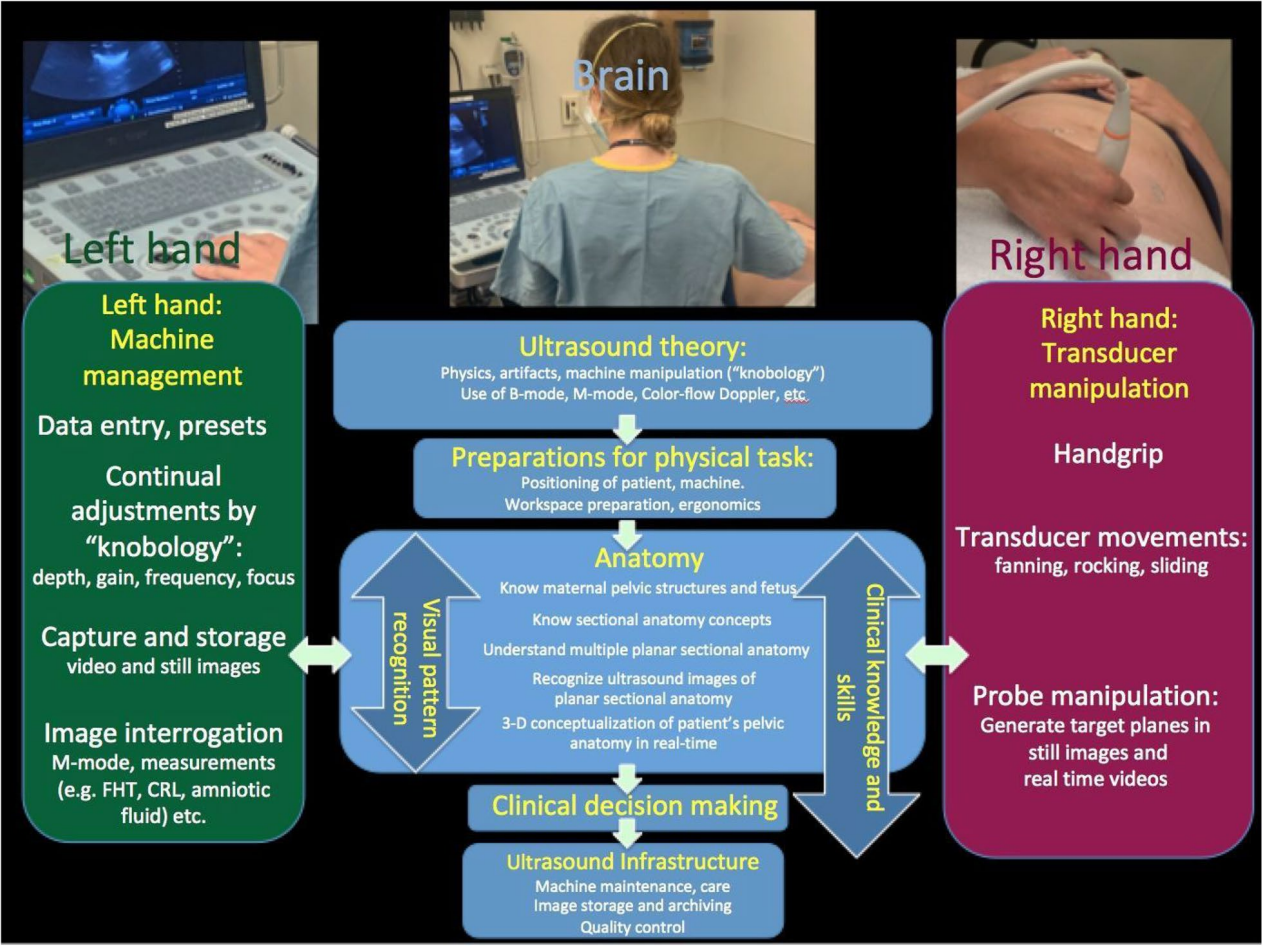
Schematic representation of the psychomotor and cognitive processes that need to be mastered to scan effectively in real-time. The schematic envisions the trainee being observed from behind, operating the machine with the left hand, and with the transducer on the patient’s skin in his/her right hand. Some sonologists may choose to reverse this right/left arrangement (scanning images courtesy of Christy Moore, RDMS)

CPUS provides valuable diagnostic information from equipment that is increasingly compact, robust, and user-friendly. Capital costs and infrastructure needs are moderate in comparison to those of other modalities of diagnostic imaging and can be used to inform clinical decisions in real-time at the point of care. A challenge is the level of educational investment that is required for the training of sonologist-clinicians. Users of CPUS must master a range of skills that include cognitive mastery of ultrasound theory, technical mastery of the ultrasound machine, anatomical knowledge, ability to conceptualize anatomy in “3-D”, psychomotor skills to manipulate an ultrasound probe with one hand while operating a machine with the other, and the ability to integrate the information obtained by ultrasound into clinical practice [36, 37]. The “muscle memory” to simultaneously operate the ultrasound machine with one hand and the transducer with the other requires extensive repetition. In addition, identification of fetal anatomy that has no uniform location or axis (in contrast, for example, to an adult heart) and normal variations in maternal habitus require complex spatial reasoning. The psychomotor processing that occurs during ultrasound scanning is summarized in Fig. 3. The importance and difficulty of acquiring these skill sets is reflected by the extensive practice requirements of most CPUS training programs. In the current study, the 6 months of on-site proctoring was deemed necessary for the relatively large group practicing in geographically disparate sites with ongoing clinical responsibilities that prevented them from using a shorter period dedicated exclusively to ultrasound training.

Our findings are consistent with previous data demonstrating that trainees can master the cognitive portion of ultrasound training [7–9, 11, 13–15, 17, 18]. Our finding that the more successful students in the OSCE were those who performed the most scans (112 compared to an average of 76.6 for those who did not pass) is open to two very different interpretations. On the one hand, it might be taken to indicate that the number of scans performed predicts successful test performance. This hypothesis would suggest that future training programs should consider a target number of required scans of 100 or more, rather than 75. However, we favor the alternative hypothesis that trainees with greater innate aptitude find it easier to learn the skills of ultrasound, and therefore perform more scans (which thereby creates a “virtuous cycle” in which they learn more from doing more scans, leading to ultrasound becoming more clinically useful to them, thereby creating a further incentive to perform more scans). This hypothesis is consistent with the common observation among those involved in ultrasound education that learners have a broad spectrum of aptitudes to the complex cognitive, visual, and psychomotor skills required to perform ultrasound [32, 33]. Based on these considerations, our finding would argue that future training programs should strive for a more tailored approach in which earlier and more frequent OSCEs would identify the trainees with natural aptitude earlier thereby allowing them to be recognized as competent for independent practice sooner, and with *less* scans. This would result in ultrasound’s benefits being conferred on the patients of the trainees with “high aptitude” while freeing up trainers to focus their time on those who need the most help. Unfortunately in the current training program, we lacked the manpower to perform more frequent OSCEs in this scattered group of learners in a geographically remote and inaccessible area.

The selection of trainees for this ultrasound course was made by the MOH with our recommended emphasis on trainees’ interest, and need for proficiency in English. Ideally, assessment of potential trainees would focus not only on interest and English proficiency, but also computer and touch-screen competency, psychomotor skills, and visuospatial intelligence. This might lead to increased successful completion of the training program, in addition to allowing for a more tailored approach to training and competency certification, as discussed above. It is also possible that teaching in learners’ native language (Swahili in this case) might accelerate learning, even among conversationally fluent English speakers.

All trainees had the same opportunities to perform proctored scans and instruction was offered outside of scheduled duty hours but many participants did not make use of this option. Conversely, trainees who successfully completed the course had higher attendance at scheduled training sessions and, if unable to attend, were more likely to schedule a make-up session. It is possible that despite these arrangements, competing personal or professional responsibilities interfered with some trainees’ ability to attend. Anecdotally we noticed that practitioners who lived far from the training sites and those with heavy clinical loads did less proctored scanning. For future training programs, it would be ideal if participants’ clinical sites could offer trainees protected time during duty hours to ensure attendance at supervised scanning sessions. Given the human and financial resource limitations in many developing-world rural areas, this will continue to be an issue when planning future courses.

An unexpected challenge for our learners, all of whom use cell phones extensively, was lack of familiarity with common components of lap-top technology such as trackballs, track-pads, keyboards, and touch screens. Before we could begin teaching image acquisition and interpretation, a significant amount of time was needed for some trainees to familiarize themselves with this interface. This slowed the learning curve that had been anticipated during the initial 10-day training course. Therefore, in future initiatives of this type, it would be useful to make a pre-course assessment of baseline familiarity with the required technology.

Our group was made up of providers with three levels of medical training. At the first level, nurse-midwives receive 2 years of tertiary (beyond secondary school) education. Clinical officers who are widely used in health systems throughout Africa receive 3 years of medical education. They work clinically by performing medical histories, physical exams, and basic treatment in health centers [38]. Further training can qualify clinical officers to perform some surgical and obstetric operations such as exploratory laparotomy and cesarean sections. Tanzanian physicians (also referred to as “medical officers” if they have not received any specialty training) have attended a 5-year medical school program followed by a mandatory 1-year general internship [38, 39]. All physicians are trained to perform routine obstetric operations. A notable finding in our study was that trainees with higher levels of education also performed better on the written and OSCE exams. These findings suggest that more extensive study of preclinical sciences (especially basic physics and general anatomy) is advantageous in developing the psychomotor skills needed in real-time ultrasound scanning. It was also noted that those with more training had better proficiency in English which may have improved communication and learning with our English-speaking group of instructors. To address these issues, we would suggest the following modifications. First, the initial pre-test should de-emphasize ultrasound knowledge (which is minimal) and try instead to identify trainees’ knowledge of anatomy, clinical management, and English proficiency. Second, basic lectures should be tailored to address knowledge gaps identified in the pre-test. Finally, time allowances should be made to teach this material in the introductory classroom course.

This study sheds new light on the challenges of ultrasound training in developing world settings, in addition to confirming some previous observations. In 2014, Swanson et al. reported on a training program for 14 midwives in rural clinics in Uganda. They successfully trained the entire group in a curriculum that was more limited than ours (cardiac activity, fetal presentation, fetal number, placental position, and first trimester identification of intrauterine pregnancy), with the stated goal of ensuring a scanning time of 5 min or less. A key element of their program was a continuous 6-week proctored course in a single location during which time trainees were relieved of all clinical responsibilities, which were taken over by temporary replacement providers. In another report of a training program for midwives, Vinayak et al*.* trained a group of 3 providers using a pre-training e-learning module followed by 4 weeks of dedicated training that included direct bedside proctoring and 2 h of lectures daily. This training period was supported by funding for trainees’ salaries, travel to a distant training location, lodging, and replacement providers to cover their clinical duties while away. The training period was followed by a period in which the scans of 271 patients were transmitted digitally for teleradiology review, with almost 100% confirmation of the midwife sonologist findings. Sonologists were able to reduce scan time from 20 to 10 min with increasing experience and confidence.

A study published in 2020 by Shah et al. examined a POCUS training protocol at labor and delivery triage for 25 Ugandan practitioners (23 midwives and 2 physicians) that included a 2-week training course followed by an additional 8 weeks of hands-on training, image quality review, and 25 proctored scans. Trainees completed OSCEs and had an 89.4% first attempt pass rate. This study benefited from having dedicated local trainers which assisted in overcoming language barriers and improved contextual understanding. Interestingly, this cohort had greater accuracy measuring femur length and more difficulty with other gestation age-related biometry measurements which is the opposite of what we found.

Our program suffered from a shorter dedicated training period (during which trainees returned to their home environments for the weekends). While it is possible that medical students whose lives are entirely dedicated to study can assimilate ultrasound skills as an integrated part of coursework, it appears that those in clinical practice (including those in residency) benefit from a period of dedicated training, which seems to lead to a more rapid mastery of ultrasound skills, improved retention, and integration of ultrasound into previously established patterns of clinical practice. To compensate for these factors, our program was designed with the continual presence of an experienced sonologist to proctor scanning and continue with didactics over a 6-month period after the initial training course.

Several other challenges were encountered in the course of our program. All of our lectures and course materials were delivered in English. Despite a requirement of English proficiency during the selection process, trainees had various degrees of English competency. Since medical school training in Tanzania is in English, this was not a problem for physicians, but was increasingly prevalent among our trainees in inverse proportion to the number of years they had spent in post-secondary school education. Future courses would ideally include a local language interpreter/trainer [22, 40]. In addition to the previously noted problem of some trainees not obtaining adequate numbers of proctored scans, 3 of the 13 trainees took leaves of absence for over a month during the training course due to illness or family circumstances. Such absences are likely to both impede psychomotor progress at the same time as eroding retention of previous training [9, 13, 41].

Common infrastructural problems can also be important impediments. Reliable electricity is often an issue in sub-Saharan Africa, leading to difficulty maintaining battery charges for the ultrasound machines. Environmental conditions with extremes of heat, humidity and dust make many parts of the developing world a challenging environment for electronic equipment, even with machines designed to military specifications such as the Sonosites used in this study. There is very limited local technical support for almost any form of medical equipment in sub-Saharan Africa, so that two (of three) ultrasound machines had to be returned to the United States for repair during the training course (one with screen, the other with battery malfunction). Since the ultrasound manufacturers do not cover the shipping for repairs, transportation of machines to and from the United States had to be arranged by the program through other groups or individuals working in Africa. Fortunately, the machines were incapacitated at different times, so that the remaining two machines could be shared between sites. However, the absence of dedicated machines at each site spanned 2 months in which trainees at one of the sites could not perform ultrasounds. We attempted to mitigate these issues by including lectures on basic maintenance and troubleshooting and would suggest that future programs make contingency plans with back-up resources for almost inevitable equipment malfunction.

The time and effort of this program depended entirely on volunteerism. While there are clear personal benefits of such work, there are also intangible as well as financial costs to the donor individuals, as well as the economic loss to the health system in the volunteers’ home country, ultimately shared by the taxpayers who help to shoulder the burden of supporting medical education [42]. Both forms of cost should be considered in ensuring that the limited resources available for medical infrastructure building in the developing world are used to the maximum effect while seeking to ensure that projects cohere with the priorities and goals of the host country [40]. The current project sought to address these competing priorities with an extensive needs assessment of the host healthcare system, and consideration of the available resources of the volunteer trainers. While it is ideal to have a trainer present continually as we did during our study period, such resources will rarely be available over such extended periods. With improving connectivity and bandwidth in all parts of the globe, it is likely that remote forms of proctoring with telemonitoring technology as well as distance learning techniques will mitigate the need for such intensive on-site supervision.

Our project was subject to challenges common to ultrasound education in remote and resource-limited settings. Limitations of manpower and the need for high trainer to trainee ratios resulted in a small cohort of learners for our study. We recognize that our small sample group limits our ability to generalize our findings to other populations or settings.

Despite the relatively low course pass rate of only 8 of 13 trainees, the MOH feels that this training has resulted in improved prenatal care in the clinics in Pemba. They have invited the PURE organization to repeat the course in the southern island of Unguja and are supporting it by applying for funding for this initiative. Along similar lines, a 1-week “train-the-trainer” course was completed in February of 2018 for the successful trainees. The impact of the “train-the-trainer” course in training a second “generation” of sonologists remains to be evaluated.

## Conclusion

A group of antenatal healthcare providers in rural Zanzibar, comprising physicians, clinical officers, and midwives, were taught basic ultrasound skills, with about 60% successfully completing the longitudinal 6-month course. Additional investigation is needed to determine trainees’ long-term retention and use of ultrasound skills, and sustainability in the form of successful trainees becoming trainers for other providers. Although the clinical impact of CPUS in the hands of providers in this setting is unknown, there is extensive evidence of its benefit in higher resource settings. Ultrasound education may lead to more widespread use of ultrasound in clinical practice and could provide similar or greater benefits in resource-limited environments where maternal fetal morbidity and mortality are much higher.

## Data Availability

The dataset supporting the conclusions of this article is available in the Zenodo repository, 10.5281/zenodo.4088929 and hyperlink to dataset: https://zenodo.org/badge/DOI/10.5281/zenodo.4088929.svg.

## Abbreviations

WHO: World Health Organization
CPUS: Clinician-performed ultrasonography
PoCUS: Point-of-care ultrasonography/point-of-care ultrasound
MOH: Ministry of Health
PURE: Point-of-care Ultrasound in Resource-limited Environments
OSCE: Objective structured clinical examination
ZAMREC: Zanzibar Medical Research and Ethics Committee
PHCU: Public health care units
PHCC: Public health care centers
EP: Emergency Practitioners
FAFF: Focused assessment for free fluid
